# Supplementary figures and images for: Schistosoma mansoni alter transcription of immunomodulatory gene products following in vivo praziquantel exposure
Source: PLoS Negl Trop Dis. 2021 Mar 3;15(3):e0009200. doi: 10.1371/journal.pntd.0009200 (PMC7959349; doi:10.1371/journal.pntd.0009200)

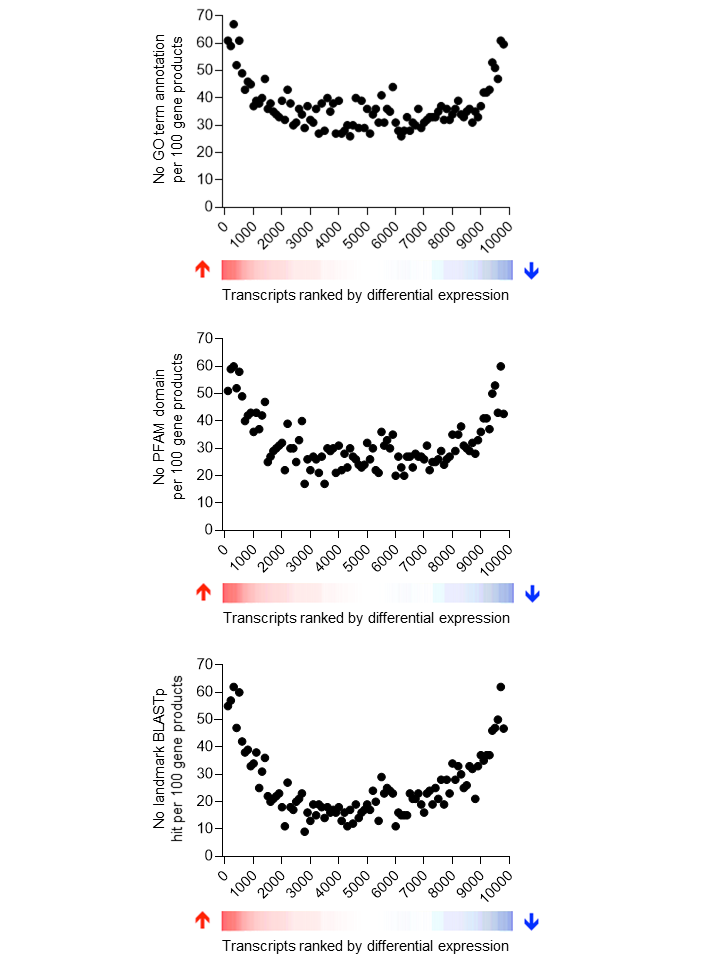

Supplement: S1 Fig — X-axis = Gene products evidenced by read mapping >0 ranked from most up-regulated to most down-regulated following PZQ treatment. Y-axis = number of transcripts that lack a GO term annotation (top), PFAM protein domain (middle) or BLASTp hit verses the landmark database (bottom) for every 100 gene products. (TIF) [file pntd.0009200.s001.tif]

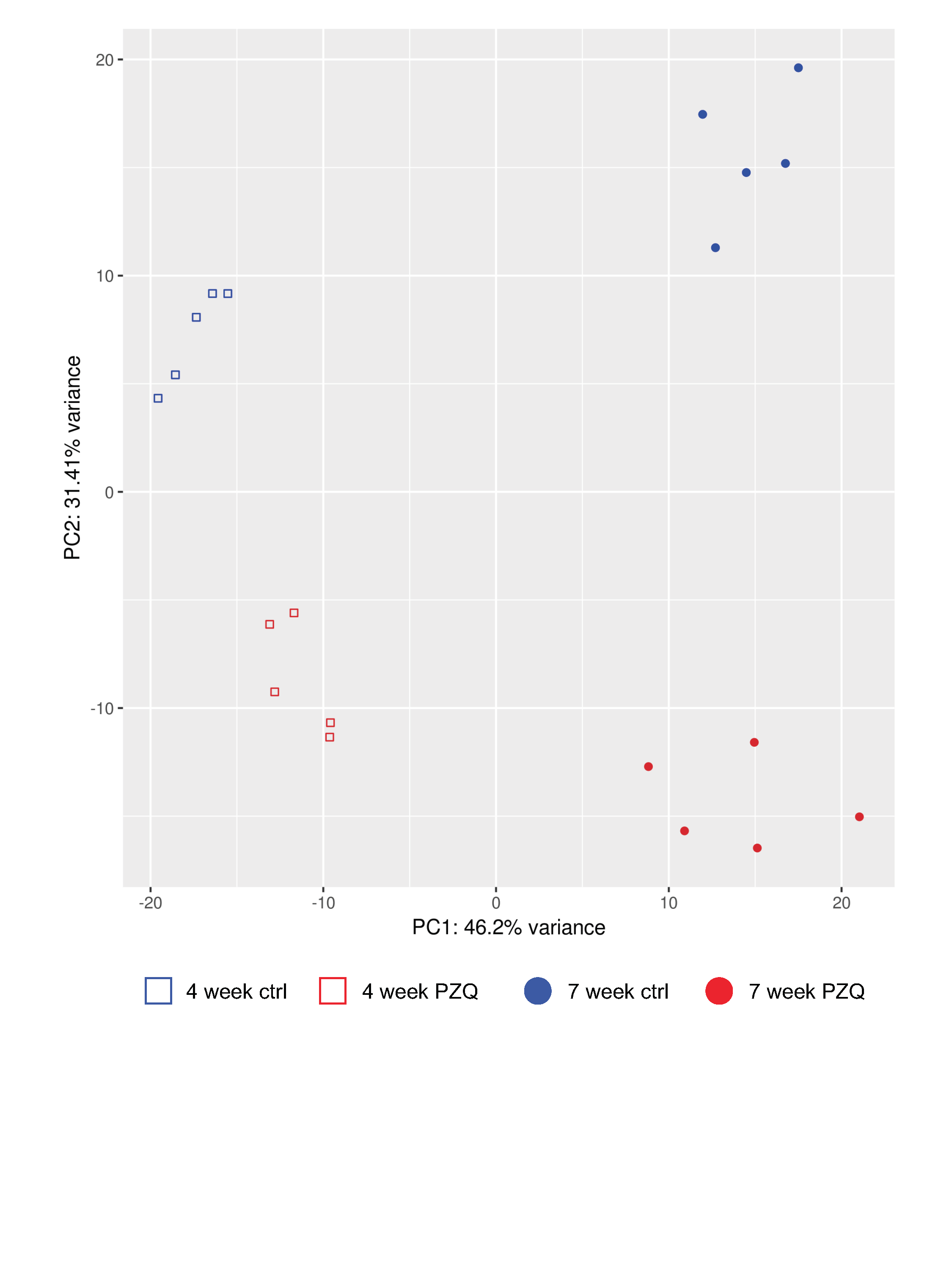

Supplement: S2 Fig — Principal component analysis was performed on RNA-Seq data from worms harvested after PZQ 100mg/kg treatment (Figs 2 and 3 and S2 and S3 Files). Blue symbols = control worms, red symbols = PZQ exposed worms. Open symbols = juvenile 4 week infections. Solid symbols = adult 7 week infections. (TIFF) [file pntd.0009200.s002.tiff]
